# Implication of Nicotinamide Adenine Dinucleotide Phosphate (NADPH) Oxidase and Its Inhibitors in Alzheimer’s Disease Murine Models

**DOI:** 10.3390/antiox10020218

**Published:** 2021-02-02

**Authors:** Leticia Guadalupe Fragoso-Morales, José Correa-Basurto, Martha Cecilia Rosales-Hernández

**Affiliations:** 1 Laboratorio de Biofísica y Biocatálisis, Escuela Superior de Medicina, Instituto Politécnico Nacional, Plan de San Luis y Díaz Mirón s/n, Mexico City 11340, Mexico; lety.23fm@gmail.com; 2 Laboratorio de Diseño y Desarrollo de Nuevos Fármacos e Innovación Biotecnológica, Escuela Superior de Medicina, Instituto Politécnico Nacional, Plan de San Luis y Díaz Mirón, Mexico City 11340, Mexico; corrjose@gmail.com

**Keywords:** Alzheimer models, NADPH oxidase, oxidative stress, NOX2, NOX4, NADPH oxidase inhibitors

## Abstract

Alzheimer’s disease (AD) is one of the main human dementias around the world which is constantly increasing every year due to several factors (age, genetics, environment, etc.) and there are no prevention or treatment options to cure it. AD is characterized by memory loss associated with oxidative stress (OS) in brain cells (neurons, astrocytes, microglia, etc.). OS can be produced by amyloid beta (Aβ) protein aggregation and its interaction with metals, mitochondrial damage and alterations between antioxidants and oxidant enzymes such as nicotinamide adenine dinucleotide phosphate (NADPH) oxidase. NADPH oxidase produces reactive oxygen species (ROS) and it is overexpressed in AD, producing large amounts of superoxide anions and hydrogen peroxide which damage brain cells and the vasculature. In addition, it has been reported that NADPH oxidase causes an imbalance of pH which could also influence in the amyloid beta (Aβ) production. Therefore, NADPH oxidase had been proposed as a therapeutic target in AD. However, there are no drugs for AD treatment such as an NADPH oxidase inhibitor despite great efforts made to stabilize the ROS production using antioxidant molecules. So, in this work, we will focus our attention on NADPH oxidase (NOX2 and NOX4) in AD as well as in AD models and later discuss the use of NADPH oxidase inhibitor compounds in AD.

## 1. Introduction

Alzheimer’s disease (AD) is a neurodegenerative disease characterized by memory loss due to multiple factors, including the generation of reactive oxygen species (ROS) [1,2]. ROS can be produced at the brain level by various pathways: a decrease in antioxidant enzymes or an increase in oxidant enzymes. One oxidant enzyme is nicotinamide adenine dinucleotide phosphate oxidase (NADPH oxidase) that increases its activity, generating more ROS, together with a decrease in antioxidant enzymes such as superoxide dismutase, catalase, etc. Additionally, alteration in the respiratory chain in the mitochondria is associated with AD due to the increased ROS. Then, all previous factors, together with an accumulation of transition metals, amyloid beta (Aβ) peptide and Tau protein could favor the generation of ROS and the progression of AD [3]. ROS can damage different biomolecules, such as lipids and proteins, and cause DNA-producing biomolecular damage capable of causing neurodegeneration that causes the cognitive deficit and memory loss of patients with AD. However, this fact could be more evident in some persons that develop sporadic AD, which results from the combination of environmental factors and genetic risk, representing more than 95% of all AD cases [4].

Despite the enormous efforts to elucidate the pathological bases of sporadic AD, these remain unclear. However, there is evidence of a relationship between AD and Aβ deposits (oligomers, fibrils, etc.) which are related to ROS production. However, several studies have been done to find a treatment for sporadic AD, even focusing on reducing ROS production or neutralizing its effect using natural antioxidants, such as carnosine, lipoic acid, *Ginkgo biloba* and curcumin [5]. To date, no treatment has been identified that prevents the course of or cures AD, since all AD treatments approved by the FDA only slow down the process, such as acetylcholinesterase inhibitors (galantamine, rivastigmine, donepezil) [6].

However, it has been identified that the generation of ROS in AD is associated with NADPH oxidase; it is overexpressed in neurons, microglia, astrocytes and cells of the cerebrovascular endothelium. Therefore, the oxidative stress (OS) generated due to ROS from the production of superoxide anions (O_2_^●−^) and hydrogen peroxide (H_2_O_2_) by NADPH oxidase is one of the main causes of cerebrovascular damage and neurodegeneration in AD [7,8].

### 1.1. Oxidative Stress Generated in the Central Nervous System

One of the main factors that can cause neurodegeneration is the imbalance between the generation of ROS and reactive nitrogen species (RNS), with the antioxidant systems (enzymatic and non-enzymatic) allowing a greater amount of ROS and RNS, thus generating OS. The production of ROS and RNS has different physiological effects since it depends on their reactivity, water solubility and also permeability in the membranes [9,10].

The generation of ROS and RNS involves a cascade of reactions that generally begins with the production of O_2_^●−^, which is of low reactivity, however, it reacts with biomolecules, increasing its chemical reactivity. O_2_^●−^ is unable to cross biological membranes due to its negative charge.

**Figure 1:**
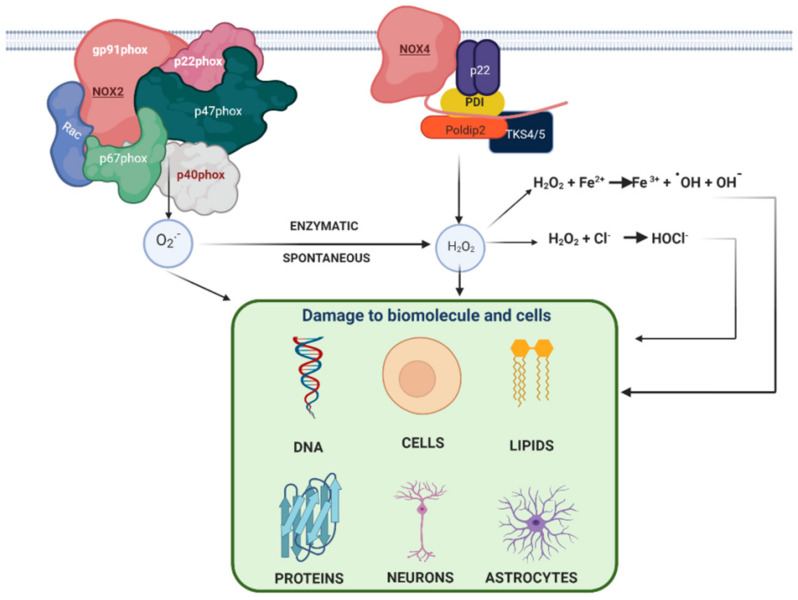
Superoxide anion (O_2_^●−^) can generate hypochlorous acid, hydroxyl radical and hydrogen peroxide which damage cells. O_2_^●−^ produced by some enzymes such as NADPH oxidase (NOX2) can be converted to H_2_O_2_ which can also be produced by NOX4. These ROS (O_2_^●−^ and H_2_O_2_) are dangerous to cells when they are overproduced.

When the O_2_^−^ is produced, it can cause damage directly or by interacting with other molecules to generate other radicals via enzymatic or non-enzymatic pathways, such as H_2_O_2_, which can react with metals to generate ROS [11,12] (Figure 1).

O_2_^●−^ is produced by the reduction of oxygen through the transfer of a free electron, and once the O_2_^●−^ is generated, it has different behavior depending on the environment conditions as O_2_^●−^ changes depending on pH, for instance. At a physiological pH, O_2_^●−^ is found in its hydroperoxyl form or perhydroxyl radical (HO_2_) which is the protonated form of O_2_^●−^ and has a great reducing capacity and can cross biological membranes [13].

In a hydrophilic environment, both O_2_^●−^ and HO_2_ can reduce ferric iron (Fe^3+^) to ferrous (Fe^2+^) which can participate in the Fenton reaction and produce hydroxyl radicals [14] (Figure 1).

Furthermore, O_2_^●−^ can be converted into hydrogen peroxide by a dismutation reaction which can be spontaneous or catalyzed by superoxide dismutase. H_2_O_2_ can easily cross membranes and react with other species, generating more reactive species, such as the during Fenton reaction (Figure 1).

During the Fenton reaction, a hydroxyl radical (**^.^**OH) is produced by the reaction between iron in the ferrous state (Fe^2+^) and H_2_O_2_, and both could be generated by the action of O_2_^●−^ OH is considered one of the most oxidizing agents as it is a very reactive free radical, and it has a short half-life, but a very high reaction rate with organic and inorganic molecules within the cell, including DNA, lipids, amino acid proteins and metals [15].

Additionally, it is important to mention that H_2_O_2_ can produce hypochlorous acid in the presence of myeloperoxidase, and hypochlorous acid is also an oxidant that can damage biomolecules such as DNA [16].

Therefore, although O_2_^●−^ could be considered a radical of low reactivity and that does not cross membranes, the products of spontaneous reaction or catalysis turn out to be very harmful for cells since the oxidizing activity of H_2_O_2_ can cause damage to cells [17,18,19,20].

OS can be generated by different pathways: from the mitochondria and derived from enzymes such as nitric oxide synthase (NOS), cytochrome P450 (cyp450), cyclooxygenase (COX), lipooxygenase, xanthine oxidase and NADPH oxidase [20].

These ROS can react with other molecules (nitric oxide), generating more intermediary, such as peroxinitrite (ONOO^●−^) [21]. However, the relationship between the ROS produced by NADPH oxidase and those generated in the mitochondria is not well known, therefore, O_2_^●−^ is produced independently by this important pathway [22].

### 1.2. Alzheimer’s Disease

AD is the most common form of human dementia. The course of the disease is characterized by slow progression, passing through mild, moderate and severe stages. Although memory deficit is a typical characteristic of the disease, patients also present problems related to cortical functions, such as aphasia, agnosia and apraxia. Furthermore, in advanced stages, AD patients suffer from depression, personality changes, anxiety and behavior problems [23].

AD can be classified as genetic and sporadic, and sporadic is the most common at 90%, compared to genetic with 10% of cases [24]. Genetic AD has been related to three genes, one related to amyloid precursor protein (APP) located on chromosome 21 and the other two related to mutations in presenilin protein 1 and 2 [25]. However, sporadic AD is more complex since genetic and environmental factors are combined [26,27]. A genetic polymorphism that is the main sporadic AD risk factor is apoliprotein E (*APOE* gene). It is highly polymorphic, and three common alleles are ε2, ε3 and ε4, which code the main isoforms of *APOE2*, *APOE3* and *APOE4*, respectively, with the ε4 allele being more associated with the risk of AD [28]. Several studies have shown an association between *APOE4* and Aβ production and Tau hyperphosphorylation [29]. Therefore, there have recently been approaches proposed for targeting *APOE* to treat AD [30].

In addition to genetic factors during sporadic AD development, there are also associated risk factors, such as hypertension, diabetes, obesity and tobacco consumption, and during the development of these risk factors, there is also an increase in ROS and, therefore, several studies have shown that OS is an important factor during the development of AD [27].

It is also important to mention that AD is most prevalent between the ages of 60 and 85 years [31]. This is the age at which the brain suffers several changes: shrinking of cortical areas, diminished neurogenesis and synaptic density, reduction in cognitive and psychomotor function and reduction in glucose metabolism in various regions, and impaired vasculature, high levels of proinflammatory cytokines and a decrease in anti-inflammatory cytokines occur [32]. In addition, with the aging, there is an increase in blood–brain barrier (BBB) permeability with which more damage by external substances could be observed [33,34]. Therefore, all physiological changes associated with age and produced by the AD development increase neurodegeneration.

AD is characterized neuropathologically by the presence of neurofibrillary tangles (NFTs), formed by the intraneuronal accumulation of Tau protein filaments and by senile plaques formed mostly by deposits of Aβ peptide [35,36]. The presence of amyloid plaques has been identified in the cortex of AD patients using staining with Thioflavin-S or Congo Red. The distribution of NFTs has been identified from the hippocampus to neocortical areas using topographic staging of NFTs [37,38].

Actually, AD diagnosis is made taking into account the cognition impairment in specific areas of brain, such as the hippocampus, cortex etc., where biomarkers are accumulated, such as Aβ and Tau [39]. To date, it is possible to determine Aβ accumulation in the brain by two methods: measure Aβ by positron emission tomography (PET) using Aβ ligands, such as Pittsburgh Compound-B (PiB), and measure the Aβ content in the cerebrospinal fluid (CSF) [40]. Additionally, Tau can be quantified in the CSF [41]. Therefore, the changes in Aβ and Tau in the CSF could be used for the diagnosis criteria of AD, however, the final diagnosis of AD still depends on postmortem neuropathological analysis.

During the formation of senile plaques and NFTs, the presence of metals has also been identified, which also contributes to ROS generation. Therefore, metals such as Fe, Cu and Zn can contribute to ROS generation by the interaction with Aβ. In addition, the generation of senile plaques causes the recruitment of glial cells which are capable of generating reactive species when interacting with Aβ since it has been reported that microglial cells contain a large amount of NADPH oxidase capable of producing O_2_^●−^ It has also been reported that NADPH oxidase is found in the vasculature of the brain, yielding high quantities of H_2_O_2_. Therefore, the generation of superoxide anions and H_2_O_2_ due to NADPH oxidase production is of great importance in the genesis and progression of AD.

### 1.3. Nicotinamide Adenine Dinucleotide Phosphate Oxidase (NADPH Oxidase) in AD

NADPH oxidase is an enzymatic complex localized in the cell membrane; in normal conditions, NADPH oxidase has many physiological roles in several proteins involved in the phosphorylation process, such as kinases and phosphatase enzymes, as well as activating transcription factors. These processes can be regulated through reduction–oxidation mechanisms [42], as NADPH oxidase is able to generate ROS as O_2_^●−^ and H_2_O_2_ [17]. These ROS can be producing depending on the NADPH oxidase isoform, and there are seven isoforms; NOX1, NOX2, NOX3, NOX4, NOX5, DUOX1 and DUOX2. Many of these isoforms have been identified in the brain, mainly in the vasculature, such as NOX1, NOX2, NOX4 and NOX5 [43].

**Figure 2:**
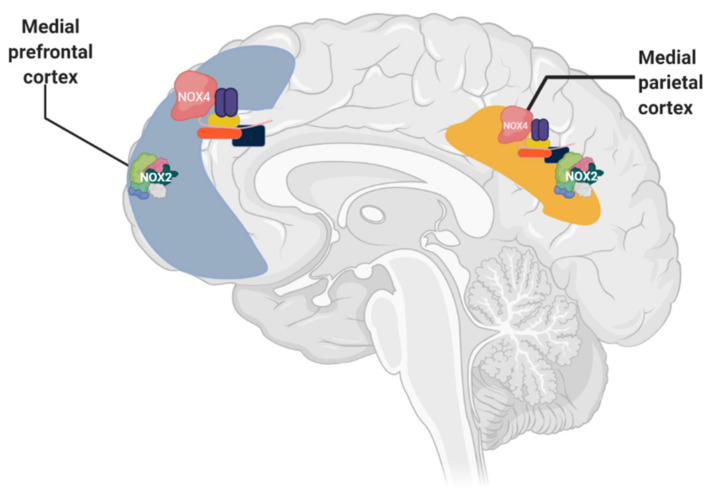
NOX2 and NOX4 isoforms identified in postmortem human brains with AD and without AD.

Therefore, NADPH oxidase is of great importance in AD as OS plays an important role in AD, and high levels of free radicals in hippocampal cells and in the cortex have been found [44,45,46]. However, not all NADPH oxidase enzymes have been identified in human brains, only in rats or mice or by using cell cultures [47]. Some of the most important isoforms identified in postmortem human brain samples with AD and without AD are NOX2 and NOX4 [47]. These enzymes can be found in the cortex region which is more implicated in Aβ production (Figure 2) and the damage during AD.

NOX2 and NOX4 have been reported to play an important role during AD, and in this review, we will focus on the role of these two NADPH oxidase isoforms in AD [48,49,50].

NOX2 is the main isoform of NADPH oxidase found in the brain in microglial cells, and these cells are considered specialized macrophages in the central nervous system [51,52]. In addition, NOX2 is found in neurons where it might be involved in the induction of neuronal cell death [53]. It has also been identified in astrocytes and in the vasculature.

**Figure 3:**
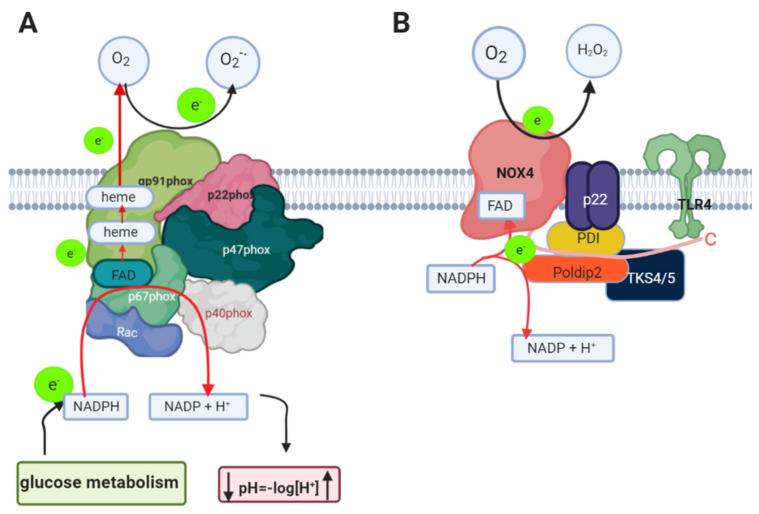
Nicotinamide adenine dinucleotide phosphate (NADPH) oxidase. (**A**) NOX2 isoform requires p67phox, p47phox, Rac and p40phox to be activated, then these cytosolic subunits need to be translocated from the cytosol to the membrane and bound to gp91phox and p22phox (membrane subunits) in order to produce the active complex. (**B**) NOX4 isoform requires protein disulfide isomerase (PDI), poldip2 and TKS4/5 for its activation, however, is not known if each one is able to activate NOX4 independently from the others. Furthermore, NOX4 can interact with Toll-like receptor 4 (TLR4).

NOX2 consists of transmembrane subunits, such as gp91phox and p22phox, and cytosolic subunits, p47phox and p67phox, and p40phox identified in phagocyte cells. Therefore, the generation of superoxide anions by NOX2 depends on the interaction between the cytosolic and the membrane subunits forming the active complex (Figure 3A) [54].

The activation of NOX2 depends on the phosphorylation of p47phox, which interacts with p22phox, facilitating their membrane translocation to interact with p67phox. In addition, Rac (a GTPase protein binding to GTP) goes to the membrane to bind to p67phox, to form the active complex (Figure 3A) [55].

Electron transfer occurs in pg91phox, which is a flavocytochrome b that contains a transmembrane domain with two heme groups, and the localization of these heme groups favors electron transfer from NADPH to FAD and the oxygen. As is shown in Figure 3A, NOX2 has cytosolic domains where NADPH and FAD bind to donate the electrons; first, NADPH transfers the electron to FAD which transfers it to the heme groups (b-type cytochromes) and finally the electron is transferred to the oxygen. The NADPH is from glucose metabolism (Figure 3A) [56,57]. Some studies show that there is displacement of the gp91phox helices that allow NADPH to bind very close to FAD [58]. Much about the NOX2 structure is known from phagocytes, however, has been proposed that NOX2 from the vasculature could have the same structure as NOX2 from phagocytes, but its regulation could be different due to its natural environment.

NOX4 is overexpressed in the endothelium of the cerebral vasculature and it has been suggested to be inducible, as when more ROS were detected, it correlated with the NOX4 mRNA [59]. NOX4 mRNA is also present in primary cultures of astrocytes [60]. The structure of NOX4 shares certain structural and functional characteristics with NOX2 (Figure 3B), for example, both depend on the interaction of p22phox in the membrane [61] and they share 39% similarity in their amino acid sequence. However, for NOX4, no cytosolic subunits are necessary for its activation as in NOX2, but it has been reported that the binding of poldip2, polymerase delta-interacting protein 2, is necessary (Figure 3B) [62]. However, although this is the most reported protein that requires NOX4 for its activation, there are others that have been reported, such as protein disulfide isomerase (PDI) [63] and a tyrosine kinase substrate with 4/5 SH3 domains (Tks4/5) [64]. Nox4 is the only isoform that produces H_2_O_2_ as a final product [65]. However, the mechanism of H_2_O_2_ production in the case of NOX4 involves O_2_^-.^ production, which seems to be trapped, and dismutase to H_2_O_2_ in an extracellular loop of NOX4, then NOX4 releases H_2_O_2_ without releasing free O_2_^●−^ [66]. It has been reported that NOX4 contains a highly conserved Hys residue which could donate protons to convert the O_2_^●−^ to H_2_O_2_ [66]. It is important to mention that these Hys residues are located in the E-loop and have not been observed in NOX1 and NOX2.

A great importance has been given to NADPH oxidase in terms of generating ROS, however, it also plays an important role as a pH modulator, because during the oxidation of NADPH, large amounts of H^+^ are produced, acidifying the intracellular environment (Figure 3). Therefore, during NADPH oxidase activity, a flux of Na^+^ ions is generated to counteract the increase in protons inside the cell and the generation of a negative charge outside the cell; this process has been well documented in neutrophils [42].

However, considering that NADPH oxidase is expressed in microglia, recent studies show that in the absence of channel protons in these cells, the H+ ions released intracellularly by NADPH oxidase are removed, then it was reported that the pH changes could be regulated by Na^+^/HCO_3_^−^ cotransporters and by Na^+^/H^+^ exchangers, therefore, these systems could be sufficient to minimize the changes in cytoplasmic pH produced by the activity of NADPH oxidase [67].

**Figure 4:**
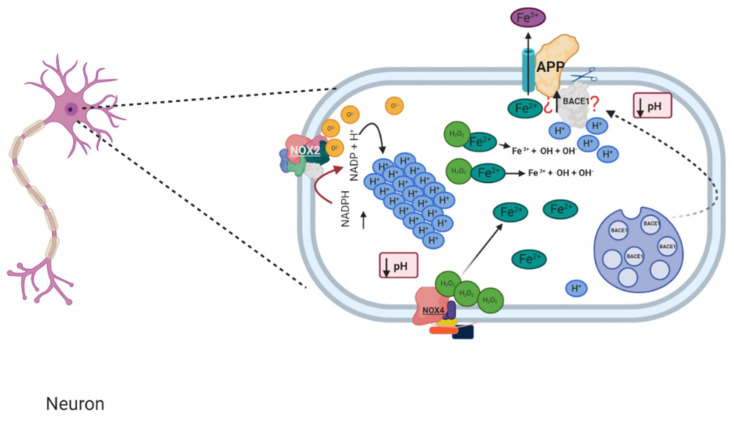
Relation between possible changes of pH induced by NADPH oxidase and BACE1 activity.

Recent studies show that NOX2 is found in neurons and astrocytes, therefore, NOX2 may play an important role in β-site APP cleaving enzyme 1 or beta-secretase 1 (BACE1) in AD patients. This is because the activity of NADPH oxidase decreases pH, possibly increasing the activity of BACE1 (Figure 4) [68]. BACE1 is localized in different cellular compartments with acidic pH; first, BACE1 is synthesized in the endoplasmic reticulum (ER) and then is distributed to the Golgi network, endosomes and cell surface, where BACE1 cleaves its cellular substrates, such as APP [69,70].

APP can be transported to the cell surface or to endosomal compartments, a process mediated by clathrin-associated vesicles. Once on the cell surface, APP can be proteolyzed by α-secretase and γ-secretase, making a complex that does not generate Aβ, which is the non-amyloidogenic pathway. However, there is other possibility that APP can be reinternalized in clathrin-coated pits in endosomal compartments containing β-secretase and the γ-secretase complex, a process by which Aβ is produced and released to the extracellular space or is degraded in lysosomes. This process is known as the amyloidogenic pathway. [71,72,73].

The promoter sites of the APP gene and several transcription factors, such as heat shock factor 1 (HSF-1) and nuclear factor kappa B (NF-κB), which are responsive to ROS, can bind here and induce APP expression [74].

Transition metals such as Fe are important catalysts for ROS production. This is due to elevated levels of iron that are implied in oxidative stress and APP production in the AD brain. There are several binding sites for redox-responsive transcription factors, such as specificity protein 1 (Sp1), NF-κB, and HIF-1α, near the promoter region of BACE1, and the expression of BACE1 may be modulated by ROS [74].

APP plays an important role in preventing iron-mediated oxidative stress through separate domains: an HO-inhibitory domain that prevents the release of Fe^2+^ from heme and, here, a separate ferroxidase domain. A failure of APP ferroxidase activity could contribute to the elevated cortical iron that characterizes AD pathology. However, ferroxidase activity of APP is unique among its protein family and, like ferritin. The ferroxidase center of APP resides in the REXXE consensus motif of the E2 domain, with a remote potentiation domain within the GDF of E1 [75,76]. Therefore, APP, together NOX, could generate more ROS production by a Fenton reaction (Figure 4).

Therefore, as NADPH oxidase releases H^+^ very close to APP, a substrate for BACE1, this could favor the generation of Aβ peptide (Figure 4). However, it has been postulated that ROS produced by NADPH oxidase directly does not influence in APP processing and it could be possible that the acid pH could influence this.

Furthermore, changing pH levels have been related to the generation and elimination of Aβ, which supports the previously described acid pH hypothesis [77]. Thus, NADPH oxidase could not only be important in AD by producing extracellular reactive species, but also by creating an intracellular pH imbalance. Due to the electrons that are transferred across, the enzyme in the membrane is depolarized and a pH imbalance on both sides of the cell membrane could be produced [42]. This event could be more severe since there are several isoforms of NADPH oxidase in brain cells, although NOX2 could be the most abundant isoform of NADPH oxidase, and it has been reported that NOX4 is also expressed in several brain regions and cells.

It is also important to mention that NADPH, a substrate of NADPH oxidase enzymes, comes from the metabolism of glucose. Glucose enters the cell to be phosphorylated, yielding glucose 6P, which can be shunted to the pentose phosphate pathway and be converted to ribulose 5P, producing NADPH [78]. Afterwards, the intermediates of the pentose phosphate pathway can be re-incorporated in the glycolysis pathway and then continue the production of NADH and its oxidation in the mitochondrial electron transport chain and oxidative phosphorylation to obtain energy in the cell [79]. The NADPH produced in the cells from the pentose phosphate pathway is of great importance because it can be used in lipid synthesis, in the detoxification of cells and also during the reduction of glutathione by the glutathione reductase enzyme [79,80]. Then, if NADPH is employed by NADPH oxidase, diminishing the reduced glutathione, this event could also to contribute to an increase in OS.

### 1.4. Relation between NADPH Oxidase and Alzheimer’s Disease

There is evidence for the participation of NADPH oxidase as one of the main sources of ROS in Alzheimer’s disease [81]. The increase in the activity and expression of NOX2 in patients with AD was observed in tissues taken postmortem, showing the translocation of the subunits (p47phox and p67phox) towards the membrane, since this form would be indicative of NADPH oxidase activation in AD, principally for NOX2 which carries out this mechanism of activation [48].

The activation and membrane translocation of NOX (subunit of p47phox and p67phox) has been demonstrated in brains (frontal lobe region) of patients with AD at 7 h postmortem, who were clinically diagnosed with AD and had an average age of 79 years [48,82]. Although the activation of NADPH oxidase could also be confirmed through its activity, the results failed to determine the source of O_2_^●−^ or H_2_O_2_ in the samples since these products can be generated from other sources and also the sample manipulation could interfere in O_2_^●−^ and H_2_O_2_ determination. These measures have been employed in systems with cells where it is necessary to employ inhibitors for other enzymes that produce these ROS as well.

Due to the above, the most reliable way to determine the activation of NADPH oxidase can be through the translocation of p47phox and p67phox for NOX2. However, for other NOX, the increased activity is associated with increased expression of some subunits, as has been determined in cultures of microglia and neurons [82].

The frontal lobe is one of the brain regions of postmortem AD patients in which the expression and activity of NADPH oxidase has been identified, such as NOX2, as well as other isoforms [83]. Other brain regions (superior and middle temporal gyri) of AD patients have also been associated with increased NOX activity, suggesting that it increased in the early stages of the disease and decreased in the later stages [48,82,84]. In rat and mouse models without or with some treatment in which the activity of NADPH oxidase is shown, the expression and activity of NOX2 and NOX4 have been reported in the hippocampus and cortex, important areas of the brain related to memory and learning [85]. In this sense, a database of an important project is Genotype–Tissue Expression (GTEx), where is possible to find gene expression and regulation, in this case for NOX2 and NOX4, where the samples are obtained from human donators. Additionally, this information is found in proteinatlas.org (Table 1), where it is possible to see that the RNA expression of NOX2 is more than that of NOX4 in the hippocampus and cortex.

Neuronal damage and death due to NOX2 upregulation has been observed, evidencing how the activation of NOX2 in the brains of patients can lead to AD [48]. However, there is evidence that NOX4 could also participate in AD due to its overexpression in astrocytes and in the vasculature. There is more evidence of the participation of NOX2 in AD, since it can be found in microglial cells that are thought to be activated through either soluble or aggregated Aβ. Microglial cells’ release of ROS, generated by the phagocyte NADPH oxidase NOX2, is thought to be a major determinant of neurotoxicity [91]. However, recently, has been reported that Aβ produces a sensitized response of Toll-like receptor 4 (TLR4) [92].

Therefore, the interaction of Aβ with TLR4 can also stimulate the NOX4 activity as a direct interaction of NOX4 through TLR4 in kidney epithelial cells (HEK293T) and U937 monocytes cells has been reported [93]. Despite not being reported in the brain, it could be a possibility for the action of Aβ in the brain due to the importance of TLR4 in microglial cells and the high TLR4 expression in AD patients’ brains [94]. Furthermore, TLR4 expression in rat hippocampal neurons increased significantly in aged neurons in vitro [95]. In addition, a specific mechanism of interaction between TLR4 and the NOX4 enzyme has been postulated, mediated by lipopolysaccharide (LPS) in endothelial cells where the TIR region of TLR4 physically interacts with the C-terminus of NOX4 [96].

It has been found that Aβ activates microglial cells and, consequently, the production of toxic and inflammatory mediators such as nitric oxide, cytokines and ROS occurs [97,98,99]. Therefore, NOX activation can be mediated by several inflammatory factors, such as Aβ, tumor necrosis factor-alpha (TNF-α), interleukins and α-synucleins, proteins also involved in AD pathology, and the activation of microglial cells and NOX can produce more inflammatory mediators, causing a vicious circle which damages the neurons and causes neuron death [100,101].

**Figure 5:**
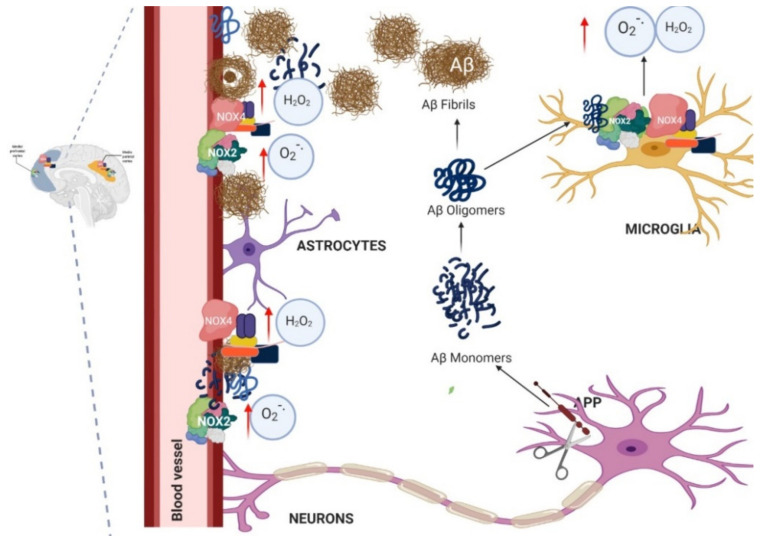
NOX2 and NOX4 in the brain and in the vasculature. NOX2 and NOX4 are the principal isoforms of NOX in the brain, and which participates in Alzheimer’s disease (AD). NOX2 and NOX4 are found in the brain cells (microglia, astrocytes, neurons, etc.) and also in endothelial cells of the vasculature in the brain. Then, when amyloid beta (Aβ) is produced from APP, the monomers and then the oligomers and fibrils are formed, and these Aβ aggregates can go to the blood vessel wall and produce neurovascular dysfunction, which is also secondary to the incomplete clearance of Aβ. In addition, the activation of NOX2 and NOX4 by Aβ oligomers can occur in brain cells to increase neurotoxins and reactive oxygen species (ROS), contributing to the neurotoxicity, cognitive deficit and dementia.

Therefore, it is important to mention that the induction and activity of NOX enzymes may be due to the large amount of Aβ. This is because Aβ can react with microglia and also astrocyte cells and induce translocation of p47phox and p67phox to the cell membrane and bind to the gp91phox subunits, activating NOX2 to initiate ROS production [96] (Figure 5). These events have been prevented by the use of apocynin, an NOX inhibitor [97]. Therefore, in many studies, it has been concluded that the OS in AD is produced by NADPH oxidase enzyme, in particular NOX2 [102].

In addition, the Aβ produced can cause effects on the cerebral vasculature [103]. It has been reported very frequently in AD patients who present with cerebral amyloid angiopathy (CAA), and this is secondary to the deposit of Aβ in the wall of the small cerebral arteries (Figure 5) [104]. There is evidence that ROS, in particular those produced by NADPH oxidase, are a key mediator of CAA-induced cardiovascular deficits [103]. Some experiments demonstrated that ROS produced by NADPH oxidase contribute to CAA pathogenesis because the use of an NADPH inhibitor reduces APOE expression levels, a factor known to promote CAA formation [25,105].

### 1.5. Murine Models of Alzheimer’s and Its Relation with NADPH Oxidase

NADPH oxidase activation, both in microglial cells and in the vasculature, is mediated by the overproduction of Aβ (Figure 5) [100]. This is of great importance since many AD models have high concentrations of Aβ, which has been considered as a key factor in the pathogenesis of AD [106,107], and which is accumulated in extracellular plaques in the brain parenchyma and in the vasculature, causing CAA. Furthermore, phosphorylated Tau accumulates, forming intraneuronal neurofibrillary tangles (NFTs), which are correlated with the severity of cognitive impairment [108]. Despite that, there is evidence to suggest that Aβ aggregates induce synaptotoxicity and neuronal death, which also correlate with cognitive deficit [109,110]. However, NADPH oxidase activation is more related to Aβ than to Tau.

Therefore, characteristics related to the production of Aβ and microglial activation and cerebrovascular damage have been identified in AD patients. Considering that Aβ plays an important role in AD, principally, it activates microglial cells and it is implicated in the generation of ROS. Several biomarkers are related to NADPH oxidase activation in microglial cells during AD, such as an increase in MHCII and CD68 markers, which indicate the activation of microglial cells [111].

Two phenotypes (pro-inflammatory, M1, and anti-inflammatory, M2) have been identified for macrophage-like microglial cells in tissues of the periphery. M1 is increased in more advanced stages of AD, while M2 is increased in early stages. However, although this classification does not seem to be sufficient to classify the activation state of microglial cells, it is a great help to identify it in AD patients in relation to its activity [112].

In relation to these states of the microglia, it has been reported that NOX2 strongly upregulated in the M1 phenotype but not in the M2 phenotype. Therefore, NOX2 participates in the M1 phenotype and neuroinflammation and contributes to neurodegeneration and the loss of neurological function [113]. Therefore, the involvement of glial cells during neurodegeneration is of great importance because NADPH oxidation could facilitate the induction of the M1 phenotype during neuroinflammation in AD [114].

In addition, patients with AD have cerebrovascular alterations attributable to the deleterious effects of Aβ on cerebral blood vessels [115] and free radical production, which is associated with alterations in vasoregulation induced by Aβ. Therefore, these effects are also diminished when a NADPH oxidase inhibitor is employed and these effects are not observed in mice without the gp91phox subunit. Thus, models that overexpress Aβ could be useful to evaluate the activity and inhibition of NADPH oxidase. Therefore, this protein is an important link between Aβ and cerebrovascular dysfunction, which could be associated with the alteration of cerebral blood flow, which occurs in AD patients [103]. Therefore, the best model of AD could be one that has all these pathological alterations [116] (Table 2).

Furthermore, more information about the transgenic mice models for the study of AD can be found at https://www.alzforum.org/research-models/alzheimers-disease. In addition, models related to alterations in Tau protein are found, such as hTau, which was designed to express only human Tau isoforms, and Tau pathology is most severe in the neocortex and hippocampus [125]. More recently, a model with the Tau mutation observed in humans was created, as the A152T MAPT mutation appears to act as a risk modifier in AD and other neurodegenerative diseases [126].

Additionally, it is important to mention that many AD models are based on mutations related to the alteration in genes for familiar AD (such as APP, PSEN1 and PSEN2) and for sporadic AD. This has been studied in non-human primates, such as great apes which accumulate Aβ in the brain and present CAA, which is correlated with Aβ, as tautopathy does not develop in great apes. It could be interesting to evaluate this in the context of NADPH oxidase activity, however, works with apes are scarce and the studies that have been done use small sample sizes. However, there are other primates, such as baboons, who develop tauopathy in the hippocampus, therefore, the results obtained depend on the non-human primates used. Many more studies have been done using Old World monkeys (e.g., rhesus monkeys, cynomolgus monkeys, baboons and vervets), and an interesting discussion about the use of these AD models is present in the review of Drummond et al. [117]. Therefore, the presence of plaques, tangles and CAA in AD models could be important to study NADPH oxidase activity and its implication in AD. Animal models (Table 2) of AD have been used to study the role of NADPH oxidase, the most widely used genetically modified model being that of Tg2576 mice. These AD models have also been genetically engineered to suppress the NOX2-/-gene and the result was a smaller amount of ROS and an improvement in cerebrovascular function and cognitive processes. In addition, this same model has been administered Aβ_1-42_ and a decrease in ROS was also observed despite the administration of Aβ [127]. Furthermore, in animal models to which Aβ_1-42_ is administered and that are genetically manipulated by deleting the p47phox-/-gene, an increase in the M2 microglia phenotype is observed. Additionally, in these animal models, a correlation between the decrease in OS with the decreased cognitive deterioration has been reported and this improvement is associated with a reduction in amyloid plaques. In addition, the use of Tg2576 mice helped to demonstrate that the vascular and behavioral effects are improved without a reduction of Aβ and amyloid plaques, indicating that the ROS produced by NOX2 did not participate in the formation of plaques [102].

### 1.6. Drugs That Could Be Used as NADPH Oxidase Inhibitors in the Treatment of AD

Since the generation of OS in AD is produced mainly by the activation of NADPH oxidase, many molecules have been evaluated as antioxidants for the treatment of the disease, but without success. Therefore, it would be more interesting to have dual molecules that can avoid NOX assembly for the case of NOX2 and also stabilize free radicals.

Many molecules fail in the treatment of AD as they do not have the same effect when passing from an animal model to humans, which is related to the use of a suitable animal model and the bioavailability of the compound that is evaluated. Very few of the NADPH oxidase inhibitors turn out to be specific for an isoform, and few of them have been evaluated in any type of AD model.

In this sense, the molecules that have been reported as NADPH oxidase inhibitors act in different ways: (a) inhibiting the interaction between the cytosolic subunits and with it the union with the membrane subunits, (b) inhibiting the expression of some subunits, (c) acting as an inhibitor of flavoprotein, (d) inhibiting some of the cytosolic components, such as Rac1 [20].

Some drugs used for the treatment of AD have been evaluated as NADPH oxidase inhibitors, such as galantamine. In a study, galantamine was evaluated in two mechanisms that produce neuronal death, one of them involving the generation of ROS (iNOS/NOX), where it was observed in hippocampal slices subjected to oxygen and glucose deprivation followed by a reoxygenation period (OGD) that galantamine produces neuroprotection by decreasing ROS production by inhibiting the NOX enzyme [128]. For other FDA drugs (except galantamine) approved for the treatment of AD, there are no data yet on whether they inhibit NADPH oxidase. However, many natural compounds have also been evaluated as NADPH oxidase inhibitors, such as apocynin, *Ginkgo biloba*, magnolol, resveratrol and blueberry-derived polyphenols [20].

Apocynin is the most studied, and it prevents p47phox and p67phox membrane translocation and, in addition, reduces the damage produced by OS by scavenging H_2_O_2_ but also acts by inhibiting other enzymes, such as rho kinases.

Another molecule widely used as an NADPH oxidase inhibitor is diphenylene iodium (DPI) and, like apocynin, it is not specific for a specific NOX isoform, and acts as general flavoprotein inhibitor and therefore inhibits endothelial nitric oxide synthase (eNOS), xanthine oxidase and proteins of the mitochondrial electron transport chain.

Nowadays, there are other NOX inhibitors which are more specific for one isoform and these last NOX inhibitors were obtained by rational drug discovery between GKT137831, ML171 and VAS2870, to name a few, which have better NOX isoform inhibition and moderate NOX isoform selectivity, and excellent work on the selectivity of the NADPH oxidase inhibitors was reported [129]. The IC_50_ values for each isoform of each NOX inhibitor, such as GKT136901, GKT137831, ML171, VAS2870, VAS3947, celastrol, ebselen, perhexiline, grindelic acid, NOX2ds-tat, NOXA1ds, fulvene-5, ACD084, phenantridinones, Shionogi and imipramin blue, are reported in [129]. Furthermore, recently, a study identified the selectivity of each NOX on different inhibitors, and it was identified that NOX1 was most potently (IC_50_) targeted by ML171 (0.1 μM); NOX2, by VAS2870 (0.7 μM); NOX4, by M13 (0.01 μM); and NOX5, by ML090(0.01 μM) [130]. However, VAS3947 was active only against NOX4 in this work reporting which compounds could be used as NOX4 inhibitors, such as pyrazoline compound, which featured significant (>40% inhibition) activity only against NOX4.

Thus, GKT136901 and GKT137831 proved to be strong NOX4 inhibitors [131]. However, some of these compounds are not specific for NOX isoforms as some of them are able to inhibit xanthine oxidase (XO), eNOS and an enzyme of mitochondrial complex 1 [132]. Many of them have been evaluated in different ways either using cell-free assays or in vivo systems.

**Figure 6:**
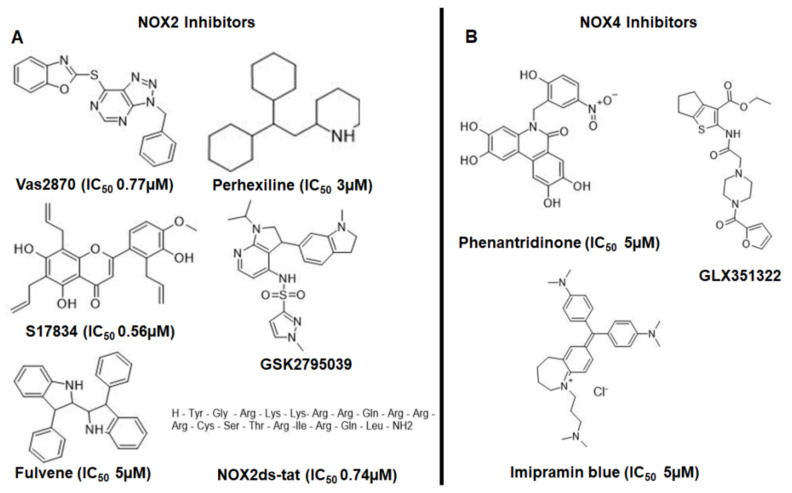
Chemical structure and IC50 values of NOX2 and NOX4 inhibitors. (**A**) Compounds with inhibitor activity to NOX2. (**B**) Compounds with inhibitor activity to NOX4.

Taking these results into account, it is possible to select those compounds with better IC50 on NOX2 and NOX4 to be evaluated in AD models (Table 2). However, the specificity of each compound is not sufficient, as one compound can inhibit a specific NOX2 or NOX4 isoform or both (Figure 6).

#### 1.6.1. VAS2870

VAS2870 was discovered by Vasopharm GmbH in a high-throughput screen for NOX2 inhibitors, and this compound was designated as a pan-NOX inhibitor [133]. A study showed that VAS2870 has better a IC_50_ value to inhibit NOX2 (IC50 ~ 0.7 μM) than NOX1 and NOX4 and, in this study, two ROS assays, Amplex Red and WST-1, were used [130]. However, controversial results have been reported on the specificity of VAS2870 and about its possible inhibitory action in cell-free assays or in cell assays, for instance, it has been cited that VAS2870 inhibits NOX2 in whole-cell lysates and as a direct NOX2 inhibitor, while another study suggests that VAS2870 showed no inhibitory activity in NOX2 and NOX5 membrane assays [134]. VAS2870 displayed low to sustained (40–70%) inhibition against NOX2 and NOX4 but not against NOX5 [131], therefore, although VAS2870 could be a good candidate to be evaluated in AD models, is cytotoxic [135].

VAS2870 has been evaluated in neurotoxicity assays with bupivacaine-induced neuronal toxicity in rats where pretreatment with VAS2870 attenuated the bupivacaine effect and it was concluded that VAS2870 diminished neuronal cell injury. This could be because VAS2870 inhibits p47phox translocation to the membrane, as it was observed that bupivacaine alone increased p47phox translocation [136].

#### 1.6.2. Perhexiline

Perhexiline is an anti-anginal drug employed in the management of patients in whom coronary revascularization is impractical [137]. Perhexiline was demonstrated to inhibit the formation of superoxide by inhibiting NOX2 with an IC_50_ of 1.5–3.6 µM, as well as in various vascular cells [128]

Perhexiline did not show scavenging ability or xanthine oxidase inhibition, which is important for its evaluation [138]. In a recent study, perhexiline was evaluated as a novel therapy in glioblastoma, and something important about this study was that perhexiline demonstrated the capacity to cross the blood–brain barrier and potent in vitro cytotoxicity [139]. The antitumor activity of perhexiline was not related directly with its ability to inhibit NOX, however, it was reported to act on tyrosine–protein kinase FYN which has been shown to interact with the NADPH oxidase NOX4 [140].

#### 1.6.3. Nox2ds-tat (gp91ds-tat)

Nox2ds-tat has been reported as a specific inhibitor of NOX2, which avoids the interaction of p47phox with gp91phox and thus inhibits ROS production by NOX2 [141]. Additionally, Nox2ds-tat has no inhibitory activity on xanthine oxidase nor has scavenger activity on superoxide. However, Nox2ds-tat was proposed to be a specific inhibitor for NOX2, and it has been reported that it is able to inhibit other NOX isoforms, such as NOX1 [142]. Nox2ds-tat has been evaluated in a traumatic brain injury (TBI) model where microglial M1 phenotype was increased. However, treatment with Nox2ds-tat 24 h after injury reduced deficits in cognitive function and increased M2-like activation in the hippocampus [113].

In a primary culture of neurons and astrocytes, NOX2ds-tat has shown a neuroprotective effect against Ca^2+^ influx, ROS production and cPLA2 and ERK1/2 produced by Aβ [143]. In addition, Nox2ds-tat could be useful in AD because it not only acts on microglial NOX, but it has also been reported that Nox2ds-tat has a functional benefit in transgenic Tg2576 AD mice, particularly in cerebral microcirculation. These include cerebral flow changes in response to Aβ protein [102,103].

However, good results have been obtained in the evaluation of Nox2ds-tat, which includes a portion of the chemical structure from compounds of the first generation (the tat portion), which was included to improve Nox2ds delivery into the cell. Several articles have reported that the inhibitory activity of gp91ds-tat is better in a cell-free system in comparison to cell system evaluation [144]. In addition, very limited oral bioavailability has been reported in several articles [145,146]. It is also not clear whether it can pass the BBB.

#### 1.6.4. GSK2795039

GSK2795039 is a novel, small molecule acting as a reversible inhibitor of NOX2 [147].

GSK2795039 had shown NOX2 inhibition after systemic administration in vivo. GSK2795039 is not cytotoxic. Although GSK2795039 was proposed as an NOX2 selective inhibitor, recently it was reported that GSK2795039 inhibits NOX2 and NOX4, with the effect being more pronounced for NOX2 (>60%) [131].

GSK2795039 prevented the effects produced by the intracerebroventricular (ICV) injection of Aβ1-42, where was identified that the main source of OS was NOX2 activation [148].

#### 1.6.5. GLX351322

GLX351322 was designed as an NOX4 inhibitor [149]. It is not cytotoxic and also has a high binding rate to plasma protein and good plasma stability, and has high permeability over biological membranes. GLX351322 does not have scavenger activity, evaluated by DPPH assay [149].

GLX351322 showed a good effect in high-fat diet (HFD)-induced glucose intolerance using male C57BL/6 mice, diminishing ROS production. This suggests that the inhibition of NOX4 was prevented by GLX351322 [149]. Recently, the use of GLX351322 in AD to study NOX4 implications during the disease has been reported.

For instance, was reported that the use of GLX351322 in APP/PS1 mice protected neurons against Aβ1-42-induced neurotoxicity, and also attenuated memory deficits and synaptic dysfunction, and finally decreased the amyloid load and OS in the hippocampus of this strain. Furthermore, Nox4-knockout APP/PS1 mice showed improved memory functions and decreased amyloid load and OS, which was consistent with the results of GLX351322. In addition, GLX351322 treatment rescued synaptic and memory deficits, and decreased OS and amyloid levels in the hippocampus of APP/PS1 mice [150].

#### 1.6.6. M13

M13 compounds from Glucox Biotech were employed in a recent study where it was identified as a first-in-class relative NOX4 selective inhibitor and 200 times more potent in inhibiting NOX4 (IC50 ~ 0.01 μM) versus NOX1 (IC50 ~ 0.2 μM) and had less activity on NOX2 and almost no effect on NOX5 [130]. Therefore, M13 could be a good selective NOX4 inhibitor to be evaluated in the damage produced by Aβ in the vasculature. No more information was found about this compound.

## 2. Conclusions

NOX2 and NOX4 are the principal isoforms of NADPH oxidase involved in AD, however, little information exists about the specific inhibitors of NADPH oxidase in AD models; many studies were done employing apocynin, a pan-NADPH inhibitor. Then, different studies could be conducted evaluating specific NOX2 and NOX4 inhibitors or the combination of both or in combination with acetylcholinesterase (AChE) inhibitors. It is now possible to obtain more selective NOX2 and NOX4 inhibitors.

The most appropriate model of AD to evaluate NOX2 and NOX4 inhibitors could be those from non-human primates who develop pathological characteristics like AD in humans. However, it could be more complicated to have these non-human primate models. Therefore, murine models which have pathological AD characteristics, such as Tg2576 and APP/PS1 mice, could be more feasible. However, few studies were found that employed these models and new NADPH oxidase inhibitors with more specificity on NOX2 and NOX4, so, in the future, the role of these molecules and their effects on these enzymes could be evaluated. In addition, with the development of new models with Tau modifications, NADPH oxidase could be studied.

## Figures and Tables

**Table 1:** Regions of the brain in rats, mice and humans in which NOX2 and NOX4 are overexpressed. * RNA expression taken from proteinatlas.org.

NADPH Oxidase Isoform	Species		
	Rat	Mouse	Human
**NOX2**	Medulla, superior colliculus, thalamus, hippocampus, hypothalamus, substantia nigra, striatum and cortex [86,87]	Thalamus, hippocampus, cerebellum, forebrain, midbrain, hindbrain, hypothalamus, substantia nigra, amygdala, striatum, cortex [86,88,89] (Cerebral cortex, olfactory region, basal ganglia, hypothalamus, thalamus, pons and medulla, hippocampus, amygdala, midbrain, retina, corpus callosum and cerebellum) *	(Cerebral cortex, olfactory region, basal ganglia, thalamus, hypothalamus, pons and medulla, hippocampus, amygdala, midbrain and cerebellum) *
**NOX4**		Hippocampus, cortex cerebellum, forebrain, midbrain, hindbrain, hypothalamus [86] (Cerebral cortex, olfactory region, basal ganglia, thalamus, hypothalamus, pons and medulla and pituitary gland) *	Brain cells [61,90] (Cerebral cortex, olfactory region, basal ganglia, hypothalamus, pons and medulla, hippocampus, amygdala, midbrain and cerebellum) *

**Table 2:** Transgenic model [117] taken from alzforum.org. * Cerebral amyloid angiopathy, ** hereditary cerebral hemorrhage with amyloidosis of the Dutch type.

	Species Model		Gene Expression			Disease	Phenotype Characterization						
	Mouse	Rat	APP	PSEN 1	MAPT		Plaques Aβ42	Tangles	Neuronal Loss	Gliosis	Synaptic Loss	Changes In LTP/LTD	Cognitive Impairment
PDAPP [118]	✓		✓	X	X	AD	✓	X	X	✓	✓	✓	✓
Tg2576 [119,120,121]	✓		✓	X	X	AD	✓	X	X	✓	✓	✓	✓
APP23 [122,123,124]	✓		✓	X	X	AD/CAA *	✓	X	✓	✓	X	X	✓
J20 (PDGF-APPSw, Ind)	✓		✓	X	X	AD	✓	X	✓	✓	✓	✓	✓
APP/PS1	✓		✓	✓	X	AD	✓	X	✓	✓	✓	✓	✓
APPswe/PS1dE9	✓		✓	✓	X	AD	✓	X	✓	✓	✓	✓	✓
Tg-SwDI	✓		✓	X	X	AD/CAA/HCH **	✓	X	Unknown	✓	Unknown	X	✓
APPE693-Δ-Tg	✓		✓	X	X	AD	X	X	✓	✓	✓	✓	✓
APP NL-G-F knock-in	✓		✓	X	X	AD	✓	X	X	✓	✓	Unknown	✓
3xTg	✓		✓	✓	✓	AD	✓	✓	Unknown	✓	X	✓	✓
5xFAD	✓		✓	✓	X	AD	✓	X	✓	✓	✓	✓	✓
PS/APP	✓		✓	✓	X	AD	✓	X	✓	✓	Unknown	Unknown	✓
TgF344-AD		✓	✓	✓	X	AD	✓	✓	✓	✓	X	X	✓
McGill-R-ThyI-APP		✓	✓	X	X	AD	✓	X	✓	✓	✓	✓	✓
